# Prognostic significance of MIB1-determined proliferative activities in intraductal components and invasive foci associated with invasive ductal breast carcinoma

**DOI:** 10.1038/sj.bjc.6690029

**Published:** 1999-09-24

**Authors:** H Imamura, S Haga, T Shimizu, O Watanabe, T Kajiwara, M Aiba

**Affiliations:** epartment of Surgery, Tokyo Women's Medical College College Daini Hospital, 2-1-10 Tokyo 116–8567; Surgical Pathology, Tokyo Women's Medical College Daini Hospital, 2-1-10, Tokyo, Arakawa-ku 116–8567, Japan

**Keywords:** invasive ductal breast carcinoma, intraductal component, invasive foci, MIB1, proliferative activity

## Abstract

The prognostic significance of the proliferative activities in intraductal components and invasive foci was investigated using 157 cases of invasive ductal breast carcinoma in which intraductal components predominated. Proliferative activity was expressed as the number of MIB1-positive nuclei per 1000 cancer cells in the most active areas of intraductal components (MLI-DCIS) or invasive foci (MLI-INV). MLI-DCIS correlated closely with MLI-INV (*r* = 0.710, 95% confidence interval, 0.623–0.780; *P*< 0.0001). Both MLI-DCIS and MLI-INV were related to oestrogen receptor (ER) (*P* = 0.0006, *P* = 0.0028 respectively), grade of invasive tumour (*P*< 0.0001, *P*< 0.0001 respectively) and classification of intraductal components (*P*< 0.0001, *P*< 0.0001 respectively). In theunivariate disease-free survival analysis, both MLI-DCIS and MLI-INV were found to be significant (*P*< 0.0001, *P* = 0.0003 respectively). However, in node-negative cases, only MLI-DCIS was significant (*P* = 0.0416). Multivariate analysis revealed that MLI-DCIS was significant not only in all cases, but also in node-negative cases (*P* = 0.0223,*P* = 0.0426 respectively), whereas MLI-INV was not. These findings indicate that MIB1-determined proliferative activity of intraductal components is a significant prognostic determinant of invasive ductal breast carcinoma in which intraductal components predominate. © 1999 Cancer Research Campaign


*British Journal of Cancer* (1999) **79**(1), 172–178
© 1999 Cancer Research Campaign

### Prognostic significance of MIB1-determined proliferative activities in intraductal components and invasive foci associated with invasive ductal breast carcinoma

**H Imamura[1], S Haga[1], T Shimizu[1], O Watanabe[1], T Kajiwara[1] and M Aiba[2]**

Departments of [1]Surgery and [2]Surgical Pathology, Tokyo Women's Medical College Daini Hospital, 2-1-10, Nishiogu, Arakawa-ku, Tokyo 116–8567, Japan

**Summary** The prognostic significance of the proliferative activities in intraductal components and invasive foci was investigated using 157 cases of invasive ductal breast carcinoma in which intraductal components predominated. Proliferative activity was expressed as the number of MIB1-positive nuclei per 1000 cancer cells in the most active areas of intraductal components (MLI-DCIS) or invasive foci (MLI-INV). MLI-DCIS correlated closely with MLI-INV ($r$ = 0.710, 95% confidence interval, 0.623–0.780; $P < 0.0001$). Both MLI-DCIS and MLI-INV were related to oestrogen receptor (ER) ($P = 0.0006$, $P = 0.0028$ respectively), grade of invasive tumour ($P < 0.0001$, $P < 0.0001$ respectively) and classification of intraductal components ($P < 0.0001$, $P < 0.0001$ respectively). In the univariate disease-free survival analysis, both MLI-DCIS and MLI-INV were found to be significant ($P < 0.0001$, $P = 0.0003$ respectively). However, in node-negative cases, only MLI-DCIS was significant ($P = 0.0416$). Multivariate analysis revealed that MLI-DCIS was significant not only in all cases, but also in node-negative cases ($P = 0.0223$, $P = 0.0426$ respectively), whereas MLI-INV was not. These findings indicate that MIB1-determined proliferative activity of intraductal components is a significant prognostic determinant of invasive ductal breast carcinoma in which intraductal components predominate.

**Keywords:** invasive ductal breast carcinoma; intraductal component; invasive foci; MIB1; proliferative activity

The proliferative activity of breast cancer cells is widely regarded as a very useful prognostic indicator. To date, numerous methods have been employed to assess proliferative activity, including counting mitotic figures (Meyer et al, 1986; Meyer, 1986; McGurrin et al, 1987; Hall and Levison, 1990; Woosley, 1991; Linden et al, 1992; Weidner et al, 1994; Collan et al, 1996), the thymidine labelling index (TLI; Meyer, 1986; Kamel et al, 1989; Silverstrini et al, 1997), the argyrophilic nucleolar organizer region (AgNOR) (Raymond and Leong, 1989; Mourad et al, 1994) and bromodeoxyuridine (BRDU; Lloveras et al, 1991; Weidner et al, 1993). Counting mitotic figures in routinely stained paraffin sections can give results comparable with more sophisticated methods of determining proliferative activity when performed by specially trained technicians (Weidner et al, 1994; Collan et al, 1996). The TLI method could provide prognostic information in breast cancers in different stages (Silverstrini et al, 1997), but it is a complex procedure requiring the use of radioisotopes. In contrast, immunohistochemical evaluation of cell-associated antigens requires neither expensive equipment nor special technical skills. This method allows preservation of architectural and cytological information and has therefore been routinely applied to a variety of tumours. The Ki-67 monoclonal antibody developed by Gerdes et al (1983) is one of the most widely used proliferation-associated markers. Recently, Cattoretti et al (1992) raised a novel antibody, called MIB1, against recombinant parts of the Ki-67 antigen. They demonstrated that the MIB1 nuclear staining seen in paraffin sections after microwave pretreatment essentially corresponds to that of Ki-67 seen in frozen sections, and some breast cancer studies have been conducted using this antibody (Barbareschi et al, 1994; Keshgegian and Cnaan, 1995; Pinder et al, 1995; Haerslev et al, 1996; Pietiläinen et al, 1996; Dettmar et al, 1997). Most earlier studies have focused on invasive foci, however, and few attempts have been made to assess the proliferative activity of malignant cells in intraductal components. As a result, the relationships between the proliferative activity of malignant cells in intraductal components and clinicopathological variables or clinical outcome have not yet been established. Recently, the biological behaviour of intraductal components has been receiving attention because extensive intraductal components have been reported to show a significant association with local recurrence after breast-conserving therapy (Bartelink et al, 1988; Kurtz et al, 1990). Moreover, early breast cancer in which intraductal components predominate is increasingly being encountered in clinical settings. In such cases, invasive foci are too small or the cancer cells are too scarce for precise evaluation of proliferative activity. In this regard, it is very important to evaluate the proliferative activity not only of invasive foci, but also of intraductal components in invasive ductal breast carcinoma. This study was designed to evaluate the proliferative activity of intraductal components associated with invasive foci in early invasive ductal breast carcinoma with predominantly intraductal components, and to examine its relationship to the proliferative activity of invasive foci. We also investigated whether the proliferative activity of intraductal components had an impact on post-operative recurrence. Before beginning the study, we decided that if it was found to have an impact we would investigate how it differed from the impact of proliferative activity in the invasive foci.

*Received 27 November 1997*
*Revised 8 April 1998*
*Accepted 16 April 1998*

*Correspondence to:* H Imamura

**Table 1** Clinicopathological variables in 157 patients with invasive ductal breast carcinoma

| Variable | Number of patients (%) |
|---|---|
| Age (years) | |
| ≤ 40 | 22 (14.0) |
| 41–65 | 113 (72.0) |
| > 65 | 22 (14.0) |
| Tumour size | |
| pT1a | 9 (5.7) |
| pT1b | 27 (17.2) |
| pT1c | 121 (77.1) |
| Axillary lymph node status | |
| Negative | 84 (53.5) |
| Positive | 73 (46.5) |
| Lymphatic vascular invasion | |
| Negative | 112 (71.3) |
| Positive | 45 (28.7) |
| ER[a] | |
| Negative | 33 (21.0) |
| Positive | 109 (69.4) |
| Unknown | 15 (9.6) |
| Grade of invasive tumour | |
| 1 | 56 (35.7) |
| 2 | 78 (49.6) |
| 3 | 23 (14.7) |
| Classification of DCIS | |
| Well differentiated | 57 (36.3) |
| Moderately | 69 (44.0) |
| Poorly | 31 (19.7) |
| Type of surgery | |
| Mastectomy | 112 (71.3) |
| Wide excision | 45 (28.7) |
| Adjuvant therapy | |
| Yes | 121 (77.1) |
| No | 36 (22.9) |
| Recurrence | |
| Yes | 36 (22.9) |
| No | 121 (77.1) |

[a]The ER determination was carried out by the EIA method with an assay kit developed by Abbott Diagnostics (Chicago, IL, USA); 13 fmol $mg^{-1}$ was taken as the minimum value of ER positivity.

### MATERIALS AND METHODS

### Patients

A total of 295 cases of UICC clinical stage I or II primary unilateral invasive ductal breast carcinoma treated at Tokyo Women's Medical College Daini Hospital between 1987 and 1993 were reviewed. To minimize the possible confounding effects of potentially inadequate pathological examination, patients who had undergone preoperative excisional biopsy, those for whom only a small number of pathology slides (ten or less) were available or those who had clinically multiple tumours were excluded. In addition, cases in which measurements of the specimen on histological sections showed diameters of invasive foci of 21 mm or more and cases in which the intraductal components were judged to be no more than 25% of the entire tumour according to the surface area ratio were excluded. The study population ultimately consisted of 157 patients. Patient and tumour characteristics are shown in Table 1. All patients were followed up for 49–128 months post-operatively, and the median follow-up was 68 months.

The majority of node-positive patients received post-operative adjuvant therapy consisting of combination chemotherapy with cyclophosphamide, methotrexate and fluorouracil (CMF) followed by oral fluorinated pyrimidine therapy. Usually, node-negative patients did not receive chemotherapy. However, if a patient was considered to be at high risk, oral fluorinated pyrimidine therapy was given. If ER status was positive or unknown, the patient also received tamoxifen therapy regardless of the type of surgery or axillary lymph node status.

Among the mastectomy patients, 30 (26.8%) experienced local recurrence and/or developed distant metastases, and 82 (73.2%) have remained disease-free to date. Among the patients receiving breast-conserving surgery, two (4.4%) experienced local recurrence, four (8.9%) developed distant metastases and 39 (86.7%) have remained disease free to date.

### Histological examination

After sampling for ER assay, the specimen was immediately fixed in buffered 10% formalin and embedded in paraffin. Tumour size was determined by direct measurement on histological sections. The cases were divided into three groups according to the diameter of the invasive focus: a pT1a group (≤ 5 mm), a pT1b group (> 5 to 10 mm) and a pT1c group (> 10 to 20 mm). In all cases, adequate numbers of permanent paraffin sections (11–69, average 24.2) were stained with haematoxylin and eosin (H&E). Every section was evaluated by two of the authors (HI and MA) without knowledge of either the immunohistochemical findings or the patient's clinical outcome.

### Grading

All invasive ductal carcinomas were graded previously on a separate occasion by two observers (HI and MA) using the method of Bloom and Richardson as modified by Elston and Ellis (1991). Intraductal components were classified by the same observers according to the classification system of ductal carcinoma in situ (DCIS; Holland et al, 1994).

### Immunohistology

After histological examination, three representative blocks from each patient were selected. All of the blocks selected were confirmed to have intraductal components associated with invasive foci. Proliferative activity was evaluated by using MIB1 antibody, as described previously (Imamura et al, 1997). After deparaffinization and dehydration, the sections were placed in 0.01 M citrate buffer at pH 6.0 for antigen retrieval. Sections were boiled five times at 95–100°C in 0.01 M citrate buffer for 3 min, and allowed to cool at room temperature. After rinsing in phosphate-buffered saline, sections were incubated for 1 h at room temperature with MIB1 antibody (Immunotech, Marseilles, France) diluted at 1:100 and immunostained using the labelled streptavidin biotin method. The complex was visualized with diaminobenzidine, and the nuclei were lightly counterstained with haematoxylin.

© Cancer Research Campaign 1999 

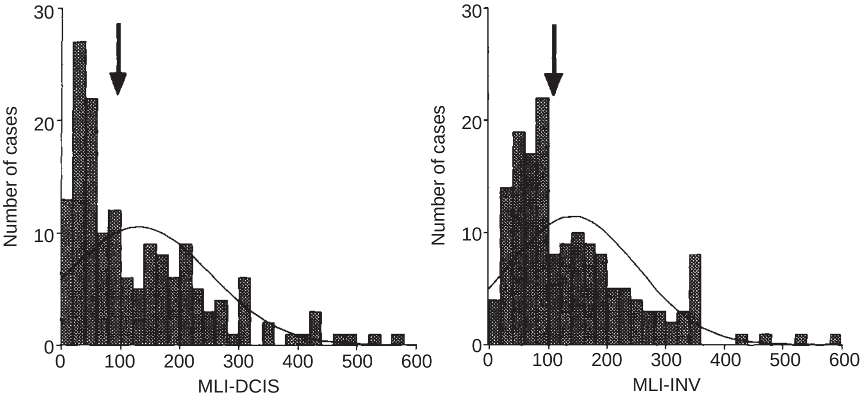

**Figure 1** Distribution of MLI-DCIS and MLI-INV in 157 breast cancers. Arrows indicate the median value of 91 in MLI-DCIS and 105 in MLI-INV

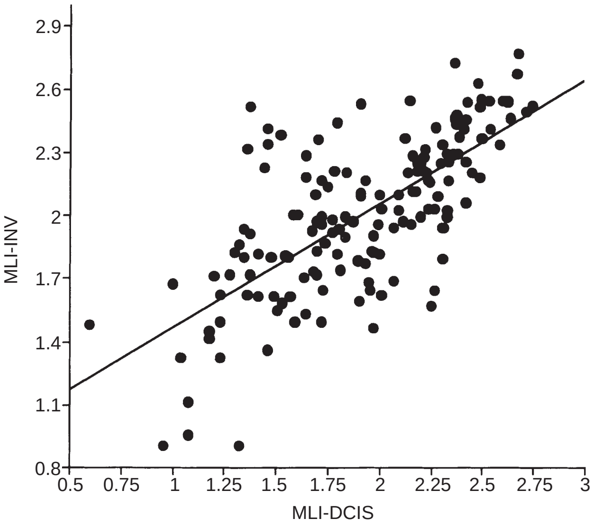

**Figure 2** Log-log plot of MLI-DCIS vs MLI-INV. The MLI-DCIS correlated highly with the MLI-INV ($r = 0.710$, 95% confidence interval, 0.623–0.780; $P < 0.0001$)

### Cell counting

All fields of the sections were scanned at low (× 40), medium (× 100) and high (× 400) power. A cell was considered positive if any nuclear staining was present. The five most strongly stained ducts were selected from the intraductal components in each tissue section to assess proliferative activity in the intraductal components. Proliferative activity in the intraductal components was expressed as the number of MIB1-positive nuclei per 1000 malignant cells viewed in high-power fields of the five ducts (MIB1 labelling index in intraductal components; MLI-DCIS). Briefly, colour photographs were taken of the five ducts selected, and more than 1000 cancer cells were counted on the photographs to calculate the number of MIB1-positive cells. Neither necrotic areas nor the edges of the ducts were included in the counting so as to minimize the possibility of immunohistochemical false positives. Likewise, proliferative activity in invasive foci was expressed as the number of MIB1-positive nuclei per 1000 malignant cells viewed in high-power fields of the most strongly stained portion of an invasive focus (MIB1 labelling index in invasive foci; MLI-INV). Usually, five colour photographs were taken of the most strongly stained portion of the invasive focus, but more than six photographs were needed for scirrhous-type invasive foci. Proliferative activity was jointly evaluated by two observers (HI and MA) who had no information on the clinical outcome of the patients.

### Statistical analysis

The associations of MLI-DCIS and MLI-INV with various clinicopathological variables were evaluated by non-parametric tests: the Mann–Whitney *U*-test for two categories, and the Kruskal–Wallis test for three categories. We used linear regression, in which measurements made by one observer (HI) were regressed against or on to those made by another observer (MA), considered to represent the standard. The correlation coefficient between MLI-DCIS and MLI-INV was analysed by Spearman's test on the log-transformed data. A value of $P < 0.05$ was considered statistically significant. Multivariate survival analysis using Cox's proportional hazard regression model was carried out to assess the independent contribution of each variable to disease free-survival. Overall survival was not analysed because of the small number of disease-related deaths (ten patients died of recurrent breast cancer). In this study, a computer program package (Stat View, Abacus Concepts, Berkeley, CA, USA) was used for all statistical testing and management of the database.

### RESULTS

### MLI-DCIS and MLI-INV in tumour specimens

Linear regression analysis revealed that the MLI-DCIS determined by HI correlated significantly with the MLI-DCIS determined by MA ($r = 0.959$; 95% confidence interval, 0.944–0.970; $P < 0.0001$). Likewise, linear regression analysis revealed that the MLI-INV determined by HI correlated significantly with the MLI-INV determined by MA ($r = 0.861$, 95% confidence interval, 0.813–0.897; $P < 0.0001$). In each case, the higher of the two independently determined MLI-DCIS and MLI-INV values, considered to reflect tumour aggressiveness most accurately, was adopted as the MLI-DCIS and MLI-INV.

Figure 1 shows the distribution of the MLI-DCIS and MLI-INV values in the 157 breast cancers. The distribution was not normal, and the median values for MLI-DCIS and MLI-INV were 91 (range 4–563) and 105 (range 8–587) respectively. As shown in Figure 2, there was a highly positive correlation between log

 © Cancer Research Campaign 1999

**Table 2** Univariate Cox analysis of disease-free survival

| | Disease-free survival | | |
|---|---|---|---|
| | **95% CI** | **RR** | ***P*** |
| *All patients (n = 157)* | | | |
| Age | 0.695–7.379 | 1.356 | 0.1404 |
| Lymphatic vascular invasion | 0.994–1.132 | 1.868 | 0.0636 |
| Axillary lymph node status | 0.096–0.464 | 3.878 | 0.0001 |
| ER | 0.353–1.850 | 0.504 | 0.6142 |
| Tumour size | 1.035–1.165 | 3.107 | 0.0019 |
| DCIS classification | 0.081–0.578 | 3.056 | 0.0093 |
| Grade of invasive tumour | 0.044–0.366 | 3.812 | 0.0005 |
| MLI-DCIS | 1.002–1.006 | 4.292 | <0.0001 |
| MLI-INV | 1.002–1.006 | 3.648 | 0.0003 |
| Type of surgery | 0.914–1.032 | 0.951 | 0.3416 |
| Adjuvant therapy | 0.364–1.904 | 0.434 | 0.6646 |
| *Node-negative patients (n = 84)* | | | |
| Age | 0.894–1.028 | 1.183 | 0.2370 |
| Lymphatic vascular invasion | 0.090–7.223 | 0.192 | 0.8479 |
| ER | 0.573–14.07 | 1.278 | 0.2013 |
| Tumour size | 0.974–1.342 | 1.641 | 0.1008 |
| DCIS classification | 0.100–3.586 | 0.561 | 0.7999 |
| Grade of invasive tumour | 0.060–7.331 | 0.334 | 0.7829 |
| MLI-DCIS | 1.000–1.008 | 2.038 | 0.0416 |
| MLI-INV | 0.995–1.007 | 0.253 | 0.8001 |
| Type of surgery | 0.109–2.677 | 0.754 | 0.4507 |
| Adjuvant therapy | 0.272–4.757 | 0.175 | 0.8607 |
| *Node-positive patients (n = 73)* | | | |
| Age | 0.538–6.000 | 1.634 | 0.1022 |
| Lymphatic vascular invasion | 0.233–1.253 | 1.435 | 0.1513 |
| ER | 0.503–2.827 | 0.399 | 0.6902 |
| Tumour size | 0.979–1.134 | 1.393 | 0.1637 |
| DCIS classification | 0.033–0.462 | 3.113 | 0.0079 |
| Grade of invasive tumour | 0.016–0.227 | 4.138 | <0.0001 |
| MLI-DCIS | 1.002–1.008 | 3.565 | 0.0004 |
| MLI-INV | 1.003–1.009 | 3.659 | 0.0003 |
| Type of surgery | 0.137–1.503 | 1.294 | 0.1597 |
| Adjuvant therapy | 0.667–5.586 | 1.213 | 0.2252 |

RR, relative risk.

MLI-DCIS and log MLI-INV ($r$ = 0.710, 95% confidence interval, 0.623–0.780; $P$ < 0.0001).

### Associations of MLI-DCIS and MLI-INV with various clinicopathological variables

MLI-DCIS correlated with three factors: ER status ($P$ = 0.0006), grade of invasive tumour ($P$ < 0.0001) and DCIS classification ($P$ < 0.0001). MLI-DCIS did not correlate with age ($P$ = 0.1794), tumour size ($P$ = 0.2514), axillary lymph node status ($P$ = 0.1097), peritumoral lymphatic vascular invasion ($P$ = 0.2745), type of surgery ($P$ = 0.0712) or adjuvant therapy ($P$ = 0.4688). MLI-INV correlated with three factors: ER status ($P$ = 0.0028), grade of invasive tumour ($P$ < 0.0001) and DCIS classification ($P$ < 0.0001). MLI-DCIS did not correlate with age ($P$ = 0.064), tumour size ($P$ = 0.4154), axillary lymph node status ($P$ = 0.0860), peritumoral lymphatic vascular invasion ($P$ = 0.9967), type of surgery ($P$ = 0.1512) or adjuvant therapy ($P$ = 0.4119).

### MLI-DCIS and MLI-INV in univariate analysis of survival

Univariate analysis focusing on disease-free survival revealed axillary lymph node status ($P$ = 0.0001), tumour size ($P$ = 0.0019), DCIS classification ($P$ = 0.0093), grade of invasive tumour ($P$ = 0.0005), MLI-DCIS ($P$ < 0.0001) and MLI-INV ($P$ = 0.0003) to be significant prognostic factors. To assess the importance of various risk factors in axillary lymph node status, univariate analysis was conducted separately for the 84 node-negative cases and the 73 node-positive cases. In the node-negative cases, only MLI-DCIS ($P$ = 0.0416) remained an important variable predicting disease-free survival. In the node-positive cases, DCIS classification ($P$ = 0.0079), grade of invasive tumour ($P$ < 0.0001), MLI-DCIS ($P$ = 0.0004) and MLI-INV ($P$ = 0.0003) remained the significant variables predicting disease-free survival (Table 2).

**Table 3** Multivariate Cox analysis of disease-free survival (model 1)

| | Disease-free survival | | |
|---|---|---|---|
| | **95% CI** | **RR** | ***P*** |
| *All patients (n = 157)* | | | |
| Axillary lymph node status | 0.108–0.543 | 3.445 | 0.0006 |
| MLI-DCIS | 1.000–1.006 | 2.285 | 0.0223 |
| DCIS classification | 0.139–1.531 | 1.264 | 0.4057 |
| Tumour size | 0.973–1.120 | 1.186 | 0.2356 |
| *Node-negative patients (n = 84)* | | | |
| MLI-DCIS | 1.000–1.017 | 2.027 | 0.0426 |
| DCIS classification | 0.286–581.7 | 1.317 | 0.4201 |
| Tumour size | 0.966–1.342 | 1.550 | 0.1212 |
| *Node-positive patients (n = 73)* | | | |
| MLI-DCIS | 0.999–1.006 | 1.649 | 0.0991 |
| DCIS classification | 0.051–1.012 | 1.994 | 0.1391 |
| Tumour size | 0.941–1.112 | 0.537 | 0.5912 |

RR, relative risk.

**Table 4** Multivariate Cox analysis of disease-free survival (model 2)

| | Disease-free survival | | |
|---|---|---|---|
| | **95% CI** | **RR** | ***P*** |
| *All patients (n = 157)* | | | |
| Axillary lymph node status | 0.101–0.502 | 3.640 | 0.0003 |
| MLI-INV | 0.999–1.005 | 1.418 | 0.1562 |
| Grade of invasive tumour | 0.049–0.529 | 3.007 | 0.0084 |
| Tumour size | 0.992–1.121 | 1.707 | 0.0878 |
| *Node-negative patients (n = 84)* | | | |
| MLI-INV | 0.994–1.007 | 0.039 | 0.9691 |
| Grade of invasive tumour | 0.051–11.04 | 0.209 | 0.7992 |
| Tumour size | 0.972–1.352 | 1.621 | 0.1051 |
| *Node-positive patients (n = 73)* | | | |
| MLI-INV | 0.999–1.007 | 1.693 | 0.0905 |
| Grade of invasive tumour | 0.024–0.457 | 2.998 | 0.0030 |
| Tumour size | 0.978–1.125 | 1.346 | 0.1784 |

RR, relative risk.

### MLI-DCIS and MLI-INV in multivariate analysis of survival

Following the univariate study, multivariate analysis was conducted to test the independent prognostic value of these variables. We took into account that the number of events was relatively few, and we selected the variables that were significant in the univariate analysis. First, in order to assess the prognostic

© Cancer Research Campaign 1999

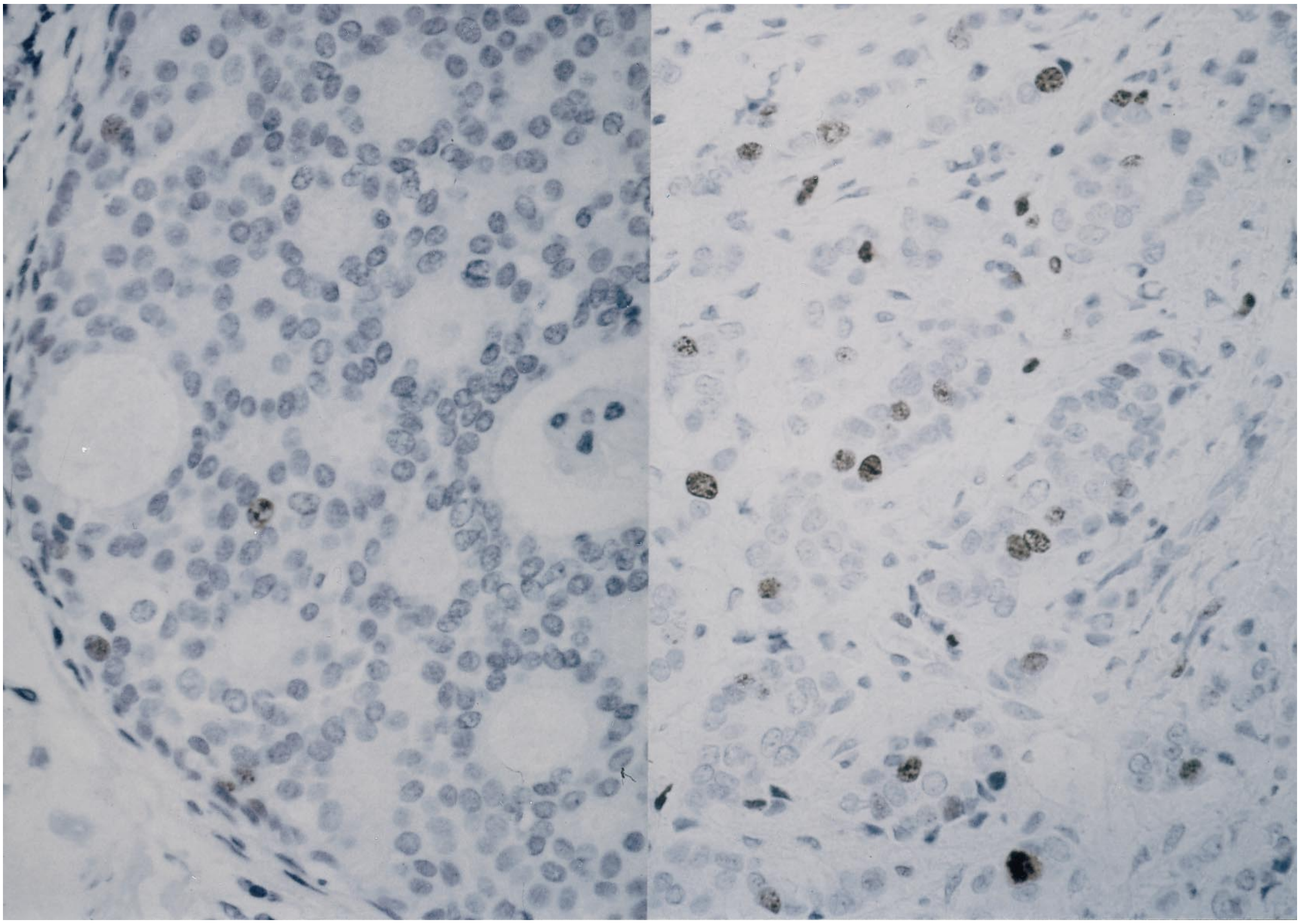

**Figure 3** Immunohistochemical features of MIB1 staining of an invasive ductal carcinoma from a 49-year-old woman. MLI-DCIS was calculated as 33 (left), and MLI-INV was calculated as 155 (right), showing marked discrepancy between the two variables (original magnification × 400)

value of intraductal components on post-operative survival, tumour size, axillary lymph node status, MLI-DCIS and DCIS classification were entered into a multivariate analysis model (model 1). The results showed that axillary lymph node status ($P$ = 0.0006) and MLI-DCIS ($P$ = 0.0223) were significant prognostic factors predicting disease-free survival. To assess the importance of variables as a risk factor for axillary lymph node status, multivariate analysis was conducted separately for the 84 node-negative cases and the 73 node-positive cases. In the node-negative cases, MLI-DCIS ($P$ = 0.0426) remained an important variable predicting disease-free survival. In the node-positive cases, no factor remained an important variable predicting disease-free survival (Table 3).

Second, in order to assess the prognostic value of invasive foci on post-operative survival, tumour size, axillary lymph node status, MLI-INV and grade of invasive tumour were entered into a multivariate analysis model (model 2). The results showed that axillary lymph node status ($P$ = 0.0003) and grade of invasive tumour ($P$ = 0.0084) were significant prognostic factors predicting disease-free survival. To assess the importance of variables as risk factors in axillary lymph node status, multivariate analysis was conducted separately for the 84 node-negative cases and the 73 node-positive cases. In the node-negative cases, no factor remained an important variable predicting disease-free survival. In the node-positive cases, only grade of invasive tumour ($P$ = 0.0030) remained a significant variable predicting disease-free survival (Table 4).

Multivariate analysis was also conducted in subgroups of patients subdivided according to surgical treatment (mastectomy vs breast-conserving surgery). The results were almost the same as in the mastectomy group, and no significant factor was found in the breast-conserving group (data not shown).

### DISCUSSION

Various prognostic factors have been investigated in invasive ductal breast carcinoma, and whereas many of the studies examined proliferative activity, most of them dealt with invasive foci (Meyer et al, 1986; Meyer, 1986; McGurrin et al, 1987; Kamel et al, 1989; Raymond and Leong, 1989; Hall and Levison, 1990; Lloveras et al, 1991; Woosley, 1991; Linden et al, 1992; Weidner et al, 1993; Mourad et al, 1994). This appears to have been because the majority of invasive ductal breast carcinoma encountered clinically in the past consisted predominantly of invasive foci and because it was impossible to distinguish between invasive foci and intraductal components by the assay methods available at the time.

*© Cancer Research Campaign 1999*

However, as DCIS and invasive ductal breast carcinoma in which the intraductal components predominate have become more common in recent years, it has become increasingly important to determine the significance of the cancer cell characteristics in the intraductal components as prognostic factors. Furthermore, as a result of advances in immunohistochemical methods, it is now possible to assay the proliferative activity of invasive foci and intraductal components separately. Clarifying the effect of the proliferative activity of intraductal components on post-operative survival is important in predicting the post-operative outcome of early breast cancer in which the intraductal components predominate. At the same time, it would also appear to provide clinical data that are useful for predicting the subsequent behaviour of residual intraductal components following breast-conserving therapy.

In the present study, there was a highly positive correlation between MLI-DCIS and MLI-INV, and, using univariate analysis of the entire group, we have also provided statistical evidence that these two variables were significant prognostic variables. However, whereas MLI-DCIS was a significant factor in the node-negative group, MLI-INV was not. Multivariate analysis indicated that MLI-DCIS is a significant prognostic determinant of disease-free survival not only in the entire group but in the node-negative group as well. Very recently, Dettmar et al (1997) studied the prognostic significance of various factors, including MIB1 proliferation rate, in 90 node-negative cases. They found that MIB1 proliferation rate was a significant prognostic factor for disease-free survival in the univariate analysis, but not in the multivariate analysis. In their study, they did not restrict subject selection to patients with DCIS-predominant breast cancer, and they did not assay the proliferative activity of invasive foci and intraductal components separately. These differences in patient selection and methods of assaying MIB1 positivity may serve as an explanation for the differences between our results and their results.

In our study, both the grade of the tumour and routine DCIS classification failed to achieve significance in the node-negative group. The grading system of Bloom and Richardson, as modified by Elston and Ellis (1991), which is widely used in routine pathology, only gives a rough estimate of the percentage of mitotic cells. Likewise, the DCIS classification system by Holland et al (1994) is convenient but overly simple, and subjective elements cannot be eliminated. Therefore, the discrepancy between results can be attributed to the varying character of these grading systems.

There are several possible reasons why MLI-INV was less significant as a prognostic factor than MLI-DCIS. One is that the fundamental biological behaviour threatening to predict recurrence of the tumour was not adequately reflected because the invasive foci were so small in our series. Another possibility is difficulty evaluating the proliferative activity of cancer cells in an invasive focus itself. More specifically, the fact that it is often difficult to differentiate between cancer cells and stromal cells when making cell counts, especially in scirrhous-type breast cancer, in which the cancer cell nests are small and the stromal cells prominent, can be cited as a possible reason for inaccurate evaluations. In contrast, differentiating cancer cells from stromal cells in intraductal components is only very rarely a problem, regardless of the subtype. There is also the advantage of it being easier to obtain accurate cell counts, because cancer cells form nests of fixed size and the cancer cells seldom overlap each other. Actually, we found some cases to possess discrepancy between MLI-DCIS and MLI-INV (Figure 3). As a result, linear correlation analysis of MLI-DCIS and MLI-INV between the two observers in the present study showed fairly high interobserver agreement, but the fact that MLI-DCIS yielded a higher interobserver agreement than MLI-INV ($r = 0.959$ vs $r = 0.861$) suggested an advantage of MLI-DCIS as a marker.

The 157 patients who served as the subjects of this study were a subset limited to 50% of the Stage I or II patients. However, when examined retrospectively, many cases had indications for breast-conserving therapy, and they are expected to increase in the future as a result of advances in imaging diagnosis. Under these circumstances, we think that showing that proliferative activity in intraductal components was significant as a prognostic factor deserves attention. We think it will be worthwhile to evaluate the relationship between the MIB1 labelling index and post-operative recurrence after breast-conserving therapy. However, in our study, the number of local recurrences after breast-conserving therapy was too small to allow determination of a meaningful correlation between the MIB1 labelling index and disease-free survival. We consider both the adequacy of excision and radiotherapy to have contributed to the favourable local control rate achieved in our patients who underwent breast-conserving therapy. There may be a complex interplay between residual intraductal components and local recurrence after breast-conserving therapy. Long-term studies involving large numbers of patients are needed to determine whether the proliferative activity of malignant cells found in intraductal components, as reflected by MIB1, correlate with local recurrence and/or patient survival following breast-conserving therapy.

The biological behaviour and clinical classification of DCIS have been attracting interest in recent years in association with advances in diagnostic imaging and increases in breast-conserving therapy. However, there has not been much interest thus far in evaluating DCIS behaviour in invasive ductal carcinoma as a prognostic factor. Recently, Gupta et al (1997) tried analysing 300 cases of invasive ductal carcinoma associated with DCIS, and stated that the morphological features of DCIS enable prediction of the clinical outcome of the patients. Expressed another way, this means that it is possible to predict the outcome of invasive ductal carcinoma early, and this is an issue that should be examined further in a larger series of cases.

In conclusion, MIB1-determined proliferative activity in intraductal components and invasive foci correlate closely, and both are predictive of disease-free survival. Furthermore, the results of multivariate analysis comparing proliferative activity in intraductal components and invasive foci showed the former to be the more important predictor, regardless of nodal status. Therefore, MIB1-determined proliferative activity in intraductal components does not simply reflect that in invasive foci, but is actually superior in terms of predicting clinical outcome of invasive ductal breast carcinoma in which intraductal components predominate, and is thus a useful tool.

### REFERENCES

Barbareschi M, Girlando S, Mauri FM, Forti S, Eccher C, Mauri FA, Togni R, Palma PD and Doglioni C (1994) Quantitative growth fraction evaluation with MIB1 and Ki67 antibodies in breast carcinomas. *Am J Clin Pathol* **102**: 171–175

Bartelink H, Borger JH, van Dongen JA and Peterse JL (1988) The impact of tumor size and histology on local control after breast-conserving therapy. *Radiother Oncol* **11**: 297–303

© Cancer Research Campaign 1999 

Cattoretti G, Becker MHG, Key G, Duchrow M, Schlüter C, Galle J and Gerdes J (1992) Monoclonal antibodies against recombinant parts of the Ki-67 antigen (MIB1 and MIB3) detect proliferating cells in microwave-processed formalin-fixed paraffin sections. *J Pathol* **168**: 357–363

Collan YUI, Kuopio T, Baak JPA, Becker R, Bogomoletz WV, Deverell M, van Diest P, van Galen C, Gilchrist K, Javed A, Kosma V-M, Kujari H, Luzi P, Mariuzzi GM, Matze E, Montironi R, Scarpelli M, Sierra D, Sisti S, Toikkanen S, Tosi P, Whimster WF and Wisse E (1996) Standardized mitotic counts in breast cancer evaluation of the method. *Pathol Res Pract* **192**: 931–941

Dettmer P, Harbeck N, Thomssen C, Pache L, Ziffer P, Fizi K, Jänicke F, Nathrath W, Schmitt M, Graeff H and Höfler H (1997) Prognostic impact of proliferation-associated factors MIB1 (Ki-67) and S-phase in node-negative breast cancer. *Br J Cancer* **75**: 1525–1533

Elston CW and Ellis IO (1991) Pathological prognostic factors in breast cancer. I. The value of histological grade in breast cancer: experience from a large study with long-term follow-up. *Histopathology* **19**: 403–410

Gerdes J, Schwab U, Lemke H and Stein H (1983) Production of a mouse monoclonal antibody reactive with a human nuclear antigen associated with cell proliferation. *Int J Cancer* **31**: 13–20

Gupta SK, Douglas-Jones AG, Fenn N, Morgan JM and Mansel RE (1997) The clinical behavior of breast carcinoma is probably determined at the preinvasive stage (ductal carcinoma in situ). *Cancer* **80**: 1740–1745

Haerslev T, Jacobsen GK and Zedeler K (1996) Correlation of growth fraction by Ki-67 and proliferating cell nuclear antigen (PCNA) immunohistochemistry with histopathological parameters and prognosis in primary breast carcinomas. *Breast Cancer Res Treat* **37**: 101–113

Hall PA and Levison DA (1990) Review: assessment of cell proliferation in histological material. *J Clin Pathol* **43**: 184–192

Holland R, Peterse JL, Millis RR, Eusebi V, Faverly D, van de Vijver MJ and Zafrani B (1994) Ductal carcinoma in situ: a proposal for a new classification. *Semin Diagn Pathol* **11**: 167–180

Imamura H, Haga S, Shimizu T, Watanabe O, Kajiwara T and Aiba M (1997) MIB1-determined proliferative activity in intraductal components and prognosis of invasive ductal breast carcinoma. *Jpn J Cancer Res* **88**: 1017–1023

Kamel OW, Franklin WA, Ringus JC and Meyer JS (1989) Thymidine labeling index and Ki-67 growth fraction in lesions of the breast. *Am J Pathol* **134**: 107–113

Keshgegian AA and Cnaan A (1995) Proliferation markers in breast carcinoma: mitotic figure count, S-phase fraction, proliferating cell nuclear antigen, Ki-67 and MIB-1. *Am J Clin Pathol* **104**: 42–49

Kurtz JM, Jacquemier J, Amalric R, Brandone H, Ayme Y, Hans D, Bressac C, Roth J and Spitalier J-M (1990) Risk factors for breast recurrence in premenopausal and postmenopausal patients with ductal cancers treated by conservation therapy. *Cancer* **65**: 1867–1878

Linden MD, Torres FX, Kubus J and Zarbo RJ (1992) Clinical application of morphologic and immunocytochemical assessments of cell proliferation. *Am J Clin Pathol (Suppl.)* **97**: 4–13

Lloveras B, Edgerton S and Thor AD (1991) Evaluation of *in vitro* bromodeoxyuridine labeling of breast carcinomas with the use of a commercial kit. *Am J Clin Pathol* **95**: 41–47

McGurrin JF, Doria MI, Dawson PJ, Karrison T, Stein HO and Franklin WA (1987) Assessment of tumor cell kinetics by immunohistochemistry in carcinoma of breast. *Cancer* **59**: 1744–1750

Meyer JS (1986) Cell kinetics of histologic variants of *in situ* breast carcinoma. *Breast Cancer Res Treat* **7**: 171–180

Meyer JS, Prey MU, Babcock DS and McDivitt RW (1986) Breast carcinoma cell kinetics, morphology, stage, and host characteristics. *Lab Invest* **54**: 41–51

Mourad WA, Setrakian S, Hales ML, Abdulla M and Trucco G (1994) The argyrophilic nucleolar organizer regions in ductal carcinoma in situ of the breast. *Cancer* **74**: 1739–1745

Pietiläinen T, Lipponen P, Aaltomaa S, Eskelinen M, Kosma V-M and Syrjänen K (1996) The important prognostic value of Ki-67 expression as determined by image analysis in breast cancer. *J Cancer Res Clin Oncol* **122**: 687–692

Pinder SE, Wencyk P, Sibbering DM, Bell JA, Elston CW, Nicholson R, Robertson JR, Blamey RW and Ellis IO (1995) Assessment of the new proliferation marker MIB1 in breast carcinoma using image analysis: associations with other prognostic factors and survival. *Br J Cancer* **71**: 146–149

Raymond WA and Leong AS-Y (1989) Nucleolar organizer regions relate to growth fractions in human breast carcinoma. *Hum Pathol* **20**: 741–746

Silvestrini R, Daidone MG, Luisi A, Mastore M, Leutner M and Sarvadori B (1997) Cell proliferation in 3,800 node-negative breast cancers: consistency over time of biological and clinical information provided by $^{3}$H-thymidine labelling index. *Int J Cancer* **74**: 122–127

Weidner N, Moore DH, Ljung B-M, Waldman FM, Goodson WH, Mayall B, Chew K and Smith HS (1993) Correlation of bromodeoxyuridine (BRDU) labeling of breast carcinoma cells with mitotic figure content and tumor grade. *Am J Surg Pathol* **17**: 987–994

Weidner N, Moore DH and Vartanian R (1994) Correlation of Ki-67 antigen expression with mitotic figure index and tumor grade in breast carcinomas using the novel ‘paraffin’-reactive MIB1 antibody. *Hum Pathol* **25**: 337–342

Woosley JT (1991) Measuring cell proliferation. *Arch Pathol Lab Med* **115**: 555–557

*© Cancer Research Campaign 1999*
